# Effect of type of disease-modifying antirheumatic drugs on depression and anxiety of patients with rheumatoid arthritis in Saudi Arabia: a cross-sectional study

**DOI:** 10.3389/fpsyt.2023.1184720

**Published:** 2023-06-06

**Authors:** Leena R. Baghdadi, Mohammed K. Alhassan, Fawaz H. Alotaibi, Anas A. Alsuwaida, Ali E. Shehadah, Mohammed T. Alzahrani

**Affiliations:** ^1^Department of Family and Community Medicine, College of Medicine, King Saud University, Riyadh, Saudi Arabia; ^2^College of Medicine, King Saud University, Riyadh, Saudi Arabia

**Keywords:** depression, anxiety, rheumatoid arthritis, DMARDs, Saudi Arabia

## Abstract

**Background:**

Rheumatoid arthritis (RA) can cause depression and anxiety. This study evaluated the factors associated with depression and anxiety in patients with RA and examined the effect of conventional and biologic disease-modifying antirheumatic drugs (DMARDs).

**Methods:**

This cross-sectional study was conducted in a regional hospital in Riyadh between March and November 2022 and included 213 patients with RA. Depression and anxiety were measured using the Hospital Anxiety and Depression Scale (HADS) and data about patients’ DMARDs use was obtained from the hospital’s medical records.

**Results:**

Based on the HADS scores, 35 (16.4%) and 49 (23%) patients with RA had depression and anxiety, respectively. There was a significant association between the level of depression and anxiety and the use of leflunomide and tocilizumab among patients with RA (*p* = 0.006 and *p* = 0.009, respectively). Patients with RA who took leflunomide had significantly higher scores for anxiety (*β* = 0.158, value of *p* = 0.037) when compared to patients who did not take leflunomide. Patients with RA who took etanercept showed a significantly lower depression score even after adjusting for confounders, including sociodemographic, clinical, and lifestyle factors (*β* = −0.189, *p* = 0.043).

**Conclusion:**

The present study highlighted the prevalence of psychiatric disorders among patients with RA and the level of depression and anxiety may differ between patients with RA depending on the type of DMARDs used. We recommend patients with RA be screened regularly for depression and anxiety to avoid further extra-articular systemic complications associated with RA.

## 1. Introduction

Rheumatoid arthritis (RA) is an inflammatory disease, which primarily targets the joints, causing joint stiffness and chronic pain, in addition to other systemic manifestations (1). These symptoms can affect the quality of life of patients with RA and limit their ambulatory capabilities and abilities, prevent them performing daily activities easily and painlessly, and these symptoms can disable their work life. These stressors can cause depression and anxiety (1), which makes these mental conditions a possibility for every patient with RA and needs prevention and treatment. Although the pathophysiology of RA has been studied extensively, a specific trigger event or cause is yet to be established. Multiple inflammatory mediators and cytokines have been associated with the disease. These factors include but are not limited to interleukin (IL)-1, IL-6, IL-8, and tumor necrosis factor alpha (TNF –α) (1). IL-6 plays a major role in the pathogenesis of RA, driving the process of synovitis and joint inflammation as well as other systemic manifestations (anemia, osteoporosis, and fatigue) (2). IL-6 is associated and mostly elevated in patients with depression and anxiety (3, 4). It gives RA the possibility of causing depression and anxiety because of the hardship and deteriorating quality of life, and as an organic cause of elevated IL-6 levels.

Rheumatoid arthritis and other chronic diseases make patients susceptible to psychiatric aspects such as depression and anxiety. Depression, anxiety, or both can lead to worse outcomes in the management of RA (5). A recent review of depression and RA conducted by Vallerand et al. reported depression can be the driving force behind RA. The review reported that the incidence rate ratio (IRR) for major depressive disorder (MDD) was significantly increased in patients with incident RA compared to the general population (IRR 1.46, 95% confidence interval [CI] 1.35–1.58) (6). The review indicates depression in patients with RA can lead to a worse course of RA and poor medication compliance (6). Matcham et al. systematically reviewed numerous studies, which estimated the prevalence of MDD in patients with RA. The systematic review and meta-analysis show a 16.8% prevalence of MDD in patients with RA (7).

In Saudi Arabia, official local databases do not estimate the prevalence of RA; therefore, information about the burden of RA is incomplete. In 2018, the Saudi Society for Rheumatology launched the Saudi Arthritis Registry, a national prospective longitudinal registry; however, no data were published (8). A study conducted over two decades ago estimated RA prevalence to be 2.2 per 1,000 of the population of Qassim, Saudi Arabia (9). A recent observational study in Saudi Arabia showed that the odds of developing depression were four times higher in patients with RA compared to patients with other chronic diseases seen in primary health care (10) and another study reported that a higher Health Assessment Questionnaire -Disability Index score was associated with higher RA activity.

Numerous pharmacologic traditional disease-modifying antirheumatic drugs (DMARDs) and new DMARDs (biologics) have been introduced in the past decades (1). These new therapies target and inhibit various immune mediators and cytokines. DMARDs exert an effect on disease activity and can affect the psychological state of the patient even though such evidence is still in the early stages. De Oliveira Ribeiro et al. compared depression and anxiety in patients with RA who were prescribed different medications and showed that treatment with biologic DMARDs (such as TNF-α antagonists) contributed to more patients with higher scores for depression when compared to patients treated with conventional DMARDs (such as methotrexate, leflunomide, and hydroxychloroquine) (11). Choy et al. showed a decrease in depression symptoms of patients started on tocilizumab (biologic DMARDs) (12). Although evidence about the effect of traditional DMARDs on psychological disorders is contradictory, some studies reported that new biologic DMARDs such as TNF-α antagonists could decrease depression symptoms in patients with RA (11). There is no conclusive evidence about the effects of DMARDs on anxiety and depression among patients with RA, especially in Saudi Arabia; therefore, we aim to examine the relationship between different groups of DMARDs and depression and anxiety scores considering clinical history, social determinants, and lifestyle factors.

## 2. Materials and methods

### 2.1. Study design and population

An analytical cross-sectional study was conducted between March and November 2022 among patients diagnosed with RA (based on the diagnosis provided by the treating physician) who were taking one or more DMARDs and attended rheumatology outpatient clinics at King Khalid University Hospital (KKUH). KKUH is a national tertiary care hospital, which accepts referrals for all age groups across Saudi Arabia and provides immediate medical access to all patients with suspected a diagnosis of rheumatoid arthritis.

Eligible participants were randomly selected and recruited from KKUH’s patient database. The electronic System for Integrated Health Information (eSiHi), which is a hospital information management system that provides patient information, including their medical notes and contact details. We used this system to search and randomly select eligible patients attending rheumatology outpatient clinics who fulfilled the inclusion criteria. We used a simple random sampling technique starting by obtaining a list of all eligible RA patients from the eSiHi. A unique identification number was assigned to each patient on the list. Then, a random number generator was used to randomly select a predetermined number of participants from the list. Those eligible patients were invited to participate in the study by filling in electronic questionnaires after giving a written informed consent.

The inclusion criteria were adult patients ≥18 years old diagnosed with RA, receiving traditional or new DMARDs (abatacept, adalimumab, certolizumab, etanercept, hydroxychloroquine, infliximab, leflunomide, methotrexate, rituximab, secukinumab, sulfasalazine, tofacitinib, and combined medications): using one of the traditional DMARDs in combination with one of the new DMARDs. The exclusion criteria were patients with malignant disease, those on high doses of methotrexate (≥20 mg per week), <18 years old, patient diagnosed with any psychiatric disorder, taking any antidepressants and anxiety medications or any other anti-psychotic medications, and RA patients who were actively using corticosteroids such as prednisolone; also, the patient’s medication chart was reviewed to make sure that eligible patients were not using any of the anti-psychotic medications or corticosteroids in the previous 3 months of the study.

### 2.2. Sample size estimation

The sample size was calculated as *n* = 209, based on the findings of a previous study that stated a 30% prevalence of depression among patients with RA who used tocilizumab (13); with a 5% margin of error and an 85% confidence interval. The sample size was calculated by the formula: $n=Z^{2}\;\left[p\left(1-p\right)\right]/d^{2}$ where,

*n* = Sample size

*Z*-score = 1.44 for confidence level 85%

*P* = expected proportion (percentage of depression)

*d* = precision (margin of error)

Substituting the above values

*N* = (1.44)^2^ [0.30 (1–0.30)]/0.05^2^

= 174 patients

The minimum sample size required was 174 participants with an additional 20% (35 participants) to compensate for potential nonresponses, incomplete data. The final sample size for the current study was calculated as 209 participants.

### 2.3. Data collection

An Arabic and English questionnaire (Supplementary Appendix A) (14, 15) was distributed electronically to eligible participants *via* links sent to their mobiles or emails listed in the patients’ hospital records. As the response rate for electronic surveys is lower than face-to-face interviews (16), a WhatsApp message reminder was sent to the participants every 3 days for approximately 2 weeks (4–5 reminders). The participants were requested to provide informed consent electronically (Supplementary Appendix B) before enrolling in the study. The patients’ medical records from eSiHi and phone calls were used to collect additional data about the patients’ medical and medication history. A pilot study was conducted on a group of 20 subjects to check the clarity and phrasing of the questionnaire. These results have not been included in this study.

The Arabic and English questionnaires had three main sections, demographic characteristics, common chronic diseases, and the HADS-A and HADS-D (Hospital Anxiety and Depression Scale) questionnaires in English and Arabic (14, 15) (Supplementary Appendix A). The first section asked for information about the patients’ characteristics, including, age, nationality, region, area of living, distance and time between home and hospital, gender, medical insurance, education level, marital status, work status, income, family composition, type of healthcare facility, and site of the injectable DMARDs. The second section asked about common chronic conditions, including hypertension, diabetes, obesity, dyslipidemia, cardiovascular disease, asthma, autoimmune disease, psychiatric illness, other chronic illnesses, in addition to lifestyle variables (weight, obesity, smoking status, physical activity, diet, alcohol consumption), medications (beta blockers, steroids, calcium channel blockers, isotretinoin, oral contraceptive pills, nonsteroidal anti-inflammatory drugs [NSAIDs]) and their adverse effect variables, and biochemical factors such as blood tests for inflammatory markers (C-reactive protein [CRP] and erythrocyte sedimentation rate [ESR]).

The third section assessed the current level of the patients’ psychological well-being, using the previously validated HADS, which is a 14-item questionnaire (14, 15). Patients were asked a series of questions about their lives and their feelings about their current situations; the answers were used to determine the patient’s level of depression and anxiety. This questionnaire has two parts, seven questions about anxiety and seven questions about depression. Each question can be answered on an ordinal 4-point scale (0 = lowest, 3 = highest). The sums of the total points from the seven responses in each section were translated into a scoring system to categorize each patient’s outcome (normal = 0–7, borderline abnormal = 8–10, abnormal = 11–21). Patients with an abnormal score in each section (anxiety and depression) (score 11–21) were considered to have depression or anxiety, respectively.

### 2.4. Data analysis

Data were analyzed using the SPSS v. 25.0. software package (SPSS, Chicago, IL, USA). Descriptive statistics (means, standard deviations, frequencies, and percentages) were used to describe the quantitative and categorical variables. Bivariate statistical analysis was performed using the student’s test and one-way analysis of variance. The Chi-square test was used to determine differences in the levels of depression and anxiety in patients by the type of DMARDs and associated factors. Statistically significant results were reported using *p* values <0.05 and 95% confidence intervals (CI). Regression analysis was conducted to examine the associations between depression, anxiety, and exposure to various DMARDs. The regression model was adjusted for potential confounders, including clinical history, inflammatory markers, exposure to medications and the use of combined DMARDs, and sociodemographic and lifestyle factors.

## 3. Results

The hospital database identified 1,993 patients who were taking one or more traditional DMARDs, and/or biologic DMARDs, including DMARDs for dermatological diseases or inflammatory bowel diseases. However, after eliminating duplicate records, only 213 patients with RA fulfilled the eligibility criteria and completed the questionnaire.

### 3.1. Sociodemographic characteristics

Table 1 shows the sociodemographic characteristics of the study’s RA population. The largest age group of participants (45.1%) was 51–65 years. Most of the patients (85.9%) were women. Almost all participants (93.9%) were Saudi nationals and lived in northern Riyadh (40%). Most patients in this study (68.1%) were married. The education level of 51.6% of the participants was a university degree and the smallest group in the education level categories was the illiterate patients (7.5%). The majority of patients with RA were unemployed (67.6%). Sixty-two participants (29.1%) earned <5,000 Saudi Riyal (SR) a month. Eighty-eight patients (43.3%) needed 16–30 min to reach the hospital from their homes, while 42 patients (19.7%) needed >1 h to reach the hospital. Although 179 (84.0%) patients had health insurance, a large number of participants (189, 88.7%) chose to have a follow-up with governmental healthcare facilities, where no private insurance is required.

**Table 1:** Demographic characteristics of the study participants (*n* = 213).

Characteristics	Subgroups	*n* = 213	%
Sociodemographic			
Age (years)	18–30	20	9.4
	31–40	34	16.0
	41–50	63	29.6
	51–65	96	45.1
Sex	Female	183	85.9
	Male	30	14.1
Nationality	Non-Saudi	13	6.1
	Saudi	200	93.9
Marital status	Single	29	13.6
	Married	145	68.1
	Divorced	19	8.9
	Widowed	20	9.4
Education	University degree	110	51.6
	High school degree	60	28.2
	Literate (able to read and write)	27	12.7
	Illiterate (unable to read or write)	16	7.5
Employment status	Employed	69	32.4
	Unemployed	144	67.6
Monthly household income (SR)	0–5,000	62	29.1
	5,001–10,000	75	35.2
	10,001–20,000	52	24.4
	>20,000	24	11.3
Living in Riyadh	Central	17	8.0
	Eastern	42	19.7
	Western	38	17.8
	Northern	85	39.9
	Southern	31	14.6
Time between home and hospital	>1 h	42	19.7
	0–15 min	30	14.1
	16–30 min	88	41.3
	31–60 min	53	24.9
Usually go to government hospital	Yes	189	88.7
	No	24	11.3
Health insurance	Yes	179	84.0
	No	34	16.0
Patients get DMARDs injections at	Government health facility	65	30.5
	Private health facility	31	14.6
	Not in a health facility	117	54.9

### 3.2. Clinical history and medications

Table 2 shows the prevalence of depression, anxiety, clinical and medication history among the study’s RA population. Based on HADS scores, 35 (16.4%) and 49 (23%) patients with RA had depression and anxiety, respectively. Thirty percent of the study population had been diagnosed with RA for 6–10 years. The patients’ most recent levels of inflammatory markers were used and showed mean levels of CRP and ESR to be 8.6 ± 18.5 μg/mL and 41.7 ± 25.6 mm/h, respectively.

**Table 2:** Distribution of depression, anxiety, clinical and medication history for patients with rheumatoid arthritis (*n* = 213).

Characteristics	Subgroups	*n* = 213	%
Depression	Normal	130	61
	Borderline abnormal	48	22.5
	Abnormal	35	16.4
Anxiety	Normal	135	63.4
	Borderline abnormal	29	13.6
	Abnormal	49	23.0
Duration of rheumatoid arthritis (years)	<= 5	38	17.8
	> 20	36	16.9
	11–15	43	20.2
	16–20	32	15.0
	6–10	64	30.0
^*^Inflammatory markers			
	C-reactive protein μg/mL	8.6 ± 18.5	
	Erythrocyte sedimentation rate mm/h	41.7 ± 25.6	
Disease-modifying antirheumatic drugs	Abatacept	4	1.9
	Adalimumab	13	6.1
	Certolizumab	7	3.3
	Etanercept	12	5.6
	Hydroxychloroquine	25	11.7
	Infliximab	1	0.5
	Leflunomide	2	0.9
	Methotrexate	56	26.3
	Rituximab	2	0.9
	Secukinumab	1	0.5
	Sulfasalazine	6	2.8
	Tocilizumab	2	0.9
	Tofacitinib	4	1.9
	^#^Combined DMARDs medication	77	36.2
Patients with comorbidities			
High blood pressure	Yes	67	31.5
	No	146	68.5
Type 2 diabetes mellitus	Yes	46	21.6
	No	167	78.4
Dyslipidemia	Yes	67	31.5
	No	146	68.5
Cardiovascular diseases	Yes	20	9.4
	No	193	90.6
Autoimmune disease	Yes	49	23.0
	No	164	77.0
Medications other than disease-modifying antirheumatic drugs			
Hypertension medication	Yes	63	29.6
	No	150	70.4
Cardiac disease medication	Yes	17	8.0
	No	196	92.0
Immunosuppressive medication	Yes	33	15.5
	No	180	84.5
Acne medication	Yes	1	0.5
	No	212	99.5
Oral contraceptive pills	Yes	12	5.6
	No	201	94.4
Nonsteroidal anti-inflammatory drugs	Yes	98	46.0
	No	115	54.0
Cancer treatment	Yes	12	5.6
	No	201	94.4

^*^Mean ± standard deviation; ^#^combined DMARDs medication: using one of the traditional DMARDs in combination with one of the new DMARDs.

Patients with RA were prescribed various conventional and biologic DMARDs. Methotrexate and hydroxychloroquine were the most used conventional DMARDS, 56 (26.3%) and 25 (11.7%) patients, respectively, exclusively used them. Around 20% of patients were prescribed only a biologic DMARD, mainly adalimumab and etanercept, and 36.2% of patients used combined RA DMARDs medication (36.2%).

Patients with RA had several chronic diseases. Sixty-seven patients (31.5%) had high blood pressure, 46 (21.6%) patients had type 2 diabetes mellitus (T2D), 67 (31.5%) patients had dyslipidemias, 20 (9.4%) patients had cardiovascular diseases, and 49 (23.0%) patients had an autoimmune disease other than RA. Patients with RA also used medications other than DMARDs. They used nonsteroidal anti-inflammatory drugs (NSAIDs) (46.0%), antihypertensive medications (29.6%), immunosuppressive medications (15.5%), cardiac disease medications (8.0%), and cancer treatments (5.6%) (Table 2).

### 3.3. Depression and anxiety and DMARDs

Table 3 compares the prevalence of depression and anxiety in patients with RA who use conventional and biological DMARDs and examines the association between patients’ levels of anxiety and depression and exposure to DMARDs. Patients with RA who used leflunomide (*p* = 0.004) showed a statistically significant difference in the proportion of depression level scores compared to patients using other DMARDs. The use of leflunomide was associated with a higher level of depression. The proportion of non-depressed patients with RA who did not take leflunomide (62.7%) was higher than patients who took leflunomide (22.2%).

**Table 3:** Comparison of depression and anxiety in patients with rheumatoid arthritis by disease-modifying antirheumatic drugs.

Disease-modifying antirheumatic drugs	Sub-group	Depression				Anxiety			
		Normal	Borderline abnormal	Abnormal	*p-*Value	Normal	Borderline abnormal	Abnormal	*p*-Value
Abatacept	Yes	5 (41.7%)	6 (50%)	1 (8.3%)	0.062	7 (58.3%)	3 (25%)	2 (16.7%)	0.476
	No	125 (62.2%)	42 (20.9%)	34 (16.9%)		128 (63.7%)	26 (12.9%)	47 (23.4%)	
Adalimumab	Yes	17 (60.7%)	5 (17.9%)	6 (21.4%)	0.669	14 (50%)	5 (17.9%)	9 (32.1%)	0.286
	No	113 (61.1%)	43 (23.2%)	29 (15.7%)		121 (65.4%)	24 (13%)	40 (21.6%)	
Certolizumab	Yes	8 (66.7%)	4 (33.3%)	0 (0%)	0.245	8 (66.7%)	2 (16.7%)	2 (16.7%)	0.848
	No	122 (60.7%)	44 (21.9%)	35 (17.4%)		127 (63.2%)	27 (13.4%)	47 (23.4%)	
Etanercept	Yes	18 (78.3%)	3 (13%)	2 (8.7%)	0.200	18 (78.3%)	2 (8.7%)	3 (13%)	0.291
	No	112 (58.9%)	45 (23.7%)	33 (17.4%)		117 (61.6%)	27 (14.2%)	46 (24.2%)	
Hydroxy-chloroquine	Yes	38 (63.3%)	13 (21.7%)	9 (15%)	0.903	39 (65%)	6 (10%)	15 (25%)	0.612
	No	92 (60.1%)	35 (22.9%)	26 (17%)		96 (62.7%)	23 (15%)	34 (22.2%)	
Infliximab	Yes	1 (33.3%)	0 (0%)	2 (66.7%)	0.05^*^	1 (33.3%)	0 (0%)	2 (66.7%)	0.186
	No	129 (61.4%)	48 (22.9%)	33 (15.7%)		134 (63.8%)	29 (13.8%)	47 (22.4%)	
Leflunomide	Yes	2 (22.2%)	2 (22.2%)	5 (55.6%)	0.004^*^	3 (33.3%)	0 (0%)	6 (66.7%)	0.006^*^
	No	128 (62.7%)	46 (22.5%)	30 (14.7%)		132 (64.7%)	29 (14.2%)	43 (21.1%)	
Methotrexate	Yes	76 (65%)	25 (21.4%)	16 (13.7%)	0.366	77 (65.8%)	14 (12%)	26 (22.2%)	0.660
	No	54 (56.3%)	23 (24%)	19 (19.8%)		58 (60.4%)	15 (15.6%)	23 (24%)	
Rituximab	Yes	6 (60%)	1 (10%)	3 (30%)	0.385	6 (60%)	1 (10%)	3 (30%)	0.842
	No	124 (61.1%)	47 (23.2%)	32 (15.8%)		129 (63.5%)	28 (13.8%)	46 (22.7%)	
Secukinumab	Yes	0 (0%)	1 (100%)	0 (0%)	0.178	1 (100%)	0 (0%)	0 (0%)	0.748
	No	130 (61.3%)	47 (22.2%)	35 (16.5%)		134 (63.2%)	29 (13.7%)	49 (23.1%)	
Sulfasalazine	Yes	4 (66.7%)	0 (0%)	2 (33.3%)	0.287	4 (66.7%)	1 (16.7%)	1 (16.7%)	0.923
	No	126 (60.9%)	48 (23.2%)	33 (15.9%)		131 (63.3%)	28 (13.5%)	48 (23.2%)	
Tocilizumab	Yes	3 (37.5%)	3 (37.5%)	2 (25%)	0.377	3 (37.5%)	4 (50%)	1 (12.5%)	0.009^*^
	No	127 (62%)	45 (22%)	33 (16.1%)		132 (64.4%)	25 (12.2%)	48 (23.4%)	
Tofacitinib	Yes	3 (75%)	0 (0%)	1 (25%)	0.540	4 (100%)	0 (0%)	0 (0%)	0.308
	No	127 (60.8%)	48 (23%)	34 (16.3%)		131 (62.7%)	29 (13.9%)	49 (23.4%)	
^#^Combined DMARDs medication	Yes	32 (66.7%)	7 (14.6%)	9 (18.8%)	0.322	30 (62.5%)	7 (14.6%)	11 (22.9%)	0.975
	No	98 (59.4%)	41 (24.8%)	26 (15.8%)		105 (63.6%)	22 (13.3%)	38 (23%)	

^*^p-Value < 0.05; ^#^combined DMARDs medication: using one of the traditional DMARDs in combination with one of the new DMARDs.

There was a significant association between the level of anxiety and the use of leflunomide and tocilizumab among patients with RA (*p* = 0.006 and *p* = 0.009, respectively). No significant associations between depression and anxiety were observed for independent or combined use of other DMARDs, including adalimumab, abatacept, etanercept, hydroxychloroquine, methotrexate, rituximab, sulfasalazine, and tofacitinib.

Supplementary Table S1 shows the results of the post-hoc test on the chi-square analysis using the pairwise probability test. The pairwise probability test was carried out by comparing the proportions of normal vs. borderline normal, normal vs. abnormal, and normal vs. abnormal borderline patients between patients who were taking certain DMARDs and those who were not. The results of pairwise comparison were in line with the results of the chi-square test where there were no differences between groups in pairs on variables that were not significantly related to levels of depression and anxiety.

However, there was a significant statistical difference between normal and abnormal score of depression (*p* = 0.004) and anxiety (*p* = 0.016) among RA patients who were taking leflunomide compared with those who were not using the medication. In addition, there was a significant association between the level of anxiety (normal vs. abnormal scores) and the use of tocilizumab among RA patients (*p* = 0.02).

### 3.4. Depression and anxiety, and clinical factors

The levels of depression and anxiety among the patients with RA were compared with exposure to clinical and medication history. The comparison showed no significant association between the levels of depression and/or anxiety and RA duration or the presence of chronic diseases (including hypertension, T2D, dyslipidemia, and cardiovascular diseases). Other autoimmune diseases showed a significant association with higher levels of depression (*p* = 0.001) but not with anxiety (*p* = 0.069); 67.7% of patients with RA who did not have autoimmune diseases were categorized as normal based on their HADS depression scores (Table 4).

**Table 4:** Comparison of depression and anxiety of rheumatoid arthritis patients by their chronic diseases.

Chronic diseases	Sub-group	Depression				Anxiety			
		Normal	Borderline abnormal	Abnormal	*p-*Value	Normal	Borderline abnormal	Abnormal	*p-*Value
Duration of rheumatoid arthritis (years)	<= 5 years	26 (68.4%)	7 (18.4%)	5 (13.2%)	0.370	22 (57.9%)	6 (15.8%)	10 (26.3%)	0.810
	6–10 years	35 (54.7%)	19 (29.7%)	10 (15.6%)		41 (64.1%)	10 (15.6%)	13 (20.3%)	
	11–15 years	29 (67.4%)	8 (18.6%)	6 (14%)		31 (72.1%)	3 (7%)	9 (20.9%)	
	16–20 years	17 (53.1%)	10 (31.3%)	5 (15.6%)		21 (65.6%)	5 (15.6%)	6 (18.8%)	
	>20 years	23 (63.9%)	4 (11.1%)	9 (25%)		20 (55.6%)	5 (13.9%)	11 (30.6%)	
Diagnosed with hypertension	Yes	42 (62.7%)	13 (19.4%)	12 (17.9%)	0.741	38 (56.7%)	9 (13.4%)	20 (29.9%)	0.262
	No	88 (60.3%)	35 (24%)	23 (15.8%)		97 (66.4%)	20 (13.7%)	29 (19.9%)	
Diagnosed with type 2 diabetes mellitus	Yes	30 (65.2%)	6 (13%)	10 (21.7%)	0.172	33 (71.7%)	2 (4.3%)	11 (23.9%)	0.113
	No	100 (59.9%)	42 (25.1%)	25 (15%)		102 (61.1%)	27 (16.2%)	38 (22.8%)	
Diagnosed with dyslipidemia	Yes	38 (56.7%)	14 (20.9%)	15 (22.4%)	0.283	38 (56.7%)	12 (17.9%)	17 (25.4%)	0.325
	No	92 (63%)	34 (23.3%)	20 (13.7%)		97 (66.4%)	17 (11.6%)	32 (21.9%)	
History of previous cardiovascular events^#^	Yes	8 (40%)	7 (35%)	5 (25%)	0.128	9 (45%)	3 (15%)	8 (40%)	0.137
	No	122 (63.2%)	41 (21.2%)	30 (15.5%)		126 (65.3%)	26 (13.5%)	41 (21.2%)	
Diagnosed with autoimmune diseases^##^	Yes	19 (38.8%)	15 (30.6%)	15 (30.6%)	0.001^*^	25 (51%)	7 (14.3%)	17 (34.7%)	0.069
	No	111 (67.7%)	33 (20.1%)	20 (12.2%)		110 (67.1%)	22 (13.4%)	32 (19.5%)	

DMARDs, disease-modifying antirheumatic drugs; ^*^p-value < 0.05; ^#^cardiovascular events (acute coronary syndrome or stroke), ^##^ autoimmune diseases (systemic auto-immune disease, organ specific autoimmune disease).

The results of the analysis in Supplementary Table S2 showed that there was no statistically significant difference in the proportion between those who had hypertension, T2D, dyslipidaemia, and cardiovascular diseases. However, significant associations with the level of depression were found in autoimmune disease showed by paired comparisons of normal vs. borderline normal depression (*p* = 0.022) and normal vs. abnormal depression (*p* = 0.001). Meanwhile, at the anxiety level of patients with autoimmune disease, there was a difference in the proportion between patients with autoimmune disease and those who did not, in the normal vs. abnormal anxiety level.

There was no significant difference in the levels of depression or anxiety between patients receiving antihypertensive or cardiac disease medications. A larger proportion of patients were either borderline depressed or abnormally depressed, if they were taking immunosuppressive drugs compared with those not taking these drugs (63.6% vs. 34.4%, *p* = 0.006). However, there was no difference in the anxiety levels between patients on immunosuppressive drugs and those not on immunosuppressive medications (*p* = 0.484). There was a significant association between the use of NSAIDs and levels of depression (*p* = 0.037) but not with levels of anxiety (*p* = 0.114) (Table 5).

**Table 5:** Comparison of depression and anxiety of rheumatoid arthritis patients by their medications (other than disease-modifying antirheumatic drugs).

Medications (other than DMARDs)	Sub-group	Depression				Anxiety			
		Normal	Borderline abnormal	Abnormal	*p-*Value	Normal	Borderline abnormal	Abnormal	*p-*Value
History of taking anti-hypertensive medication^#^	Yes	40 (63.5%)	11 (17.5%)	12 (19%)	0.476	34 (54%)	9 (14.3%)	20 (31.7%)	0.119
	No	90 (60%)	37 (24.7%)	23 (15.3%)		101 (67.3%)	20 (13.3%)	29 (19.3%)	
History of taking cardiac disease medication^##^	Yes	10 (58.8%)	5 (29.4%)	2 (11.8%)	0.723	10 (58.8%)	3 (17.6%)	4 (23.5%)	0.868
	No	120 (61.2%)	43 (21.9%)	33 (16.8%)		125 (63.8%)	26 (13.3%)	45 (23%)	
History of taking immuno-suppressive medication^###^	Yes	12 (36.4%)	13 (39.4%)	8 (24.2%)	0.006^*^	18 (54.5%)	5 (15.2%)	10 (30.3%)	0.484
	No	118 (65.6%)	35 (19.4%)	27 (15%)		117 (65%)	24 (13.3%)	39 (21.7%)	
History of taking acne medication^$^	Yes	0 (0%)	1 (100%)	0 (0%)	0.178	0 (0%)	0 (0%)	1 (100%)	0.186
	No	130 (61.3%)	47 (22.2%)	35 (16.5%)		135 (63.7%)	29 (13.7%)	48 (22.6%)	
History of taking oral contraceptive pills	Yes	7 (58.3%)	3 (25%)	2 (16.7%)	0.975	6 (50%)	3 (25%)	3 (25%)	0.451
	No	123 (61.2%)	45 (22.4%)	33 (16.4%)		129 (64.2%)	26 (12.9%)	46 (22.9%)	
Nonsteroidal anti-inflammatory drugs	Yes	54 (55.1%)	21 (21.4%)	23 (23.5%)	0.037^*^	55 (56.1%)	15 (15.3%)	28 (28.6%)	0.114
	No	76 (66.1%)	27 (23.5%)	12 (10.4%)		80 (69.6%)	14 (12.2%)	21 (18.3%)	
History of taking cancer treatment^¥^	Yes	6 (50%)	3 (25%)	3 (25%)	0.652	6 (50%)	1 (8.3%)	5 (41.7%)	0.280
	No	124 (61.7%)	45 (22.4%)	32 (15.9%)		129 (64.2%)	28 (13.9%)	44 (21.9%)	

DMARDs, disease-modifying antirheumatic drugs; ^*^p-value < 0.05; ^#^anti-hypertensive medications (angiotensin-converting enzyme inhibitors, angiotensin receptor blockers, beta blocker, calcium channel blockers and diuretics); ^##^cardiac disease medication (anticoagulants, antiplatelets, and antiarrhythmic drugs); ^$^oral contraceptive pills (combined estrogen-progesterone, and progesterone-only); ^¥^cancer treatment (chemotherapy, radiation therapy, immunotherapy, and targeted therapy); ^###^immuno-suppressive medications (glucocorticoids, tacrolimus, azathioprine, mycophenolate mofetil, and cyclophosphamide).

Supplementary Table S3 shows that there was a significant difference in the comparison of normal vs. borderline normal depression in patients with immunosuppressive drug (*p* = 0.005). Similar results were also found in the comparison of the proportion of normal vs. abnormal depression levels in patients using NSAIDs (*p* = 0.019).

These factors were adjusted in the regression models to eliminate their effects on the association between exposure to DMARDs and depression and anxiety scores.

### 3.5. Depression and anxiety in DMARDs exposure and clinical, sociodemographic, and lifestyle factors

Unadjusted (Model 1) and adjusted results (Model 2 adjusted for the use of combined DMARDs, inflammatory markers, and sociodemographic lifestyle factors) for anxiety and depression comparing DMARDs groups are presented in Tables 6, 7. In the overall unadjusted analysis, patients with RA taking leflunomide had significantly higher scores for anxiety (*β* = 0.158, value of *p* = 0.037) compared to the patients who did not take leflunomide (Table 6). After adjusting for the use of combined DMARDs, inflammatory markers, and sociodemographic and lifestyle factors, the patients with RA treated with leflunomide still showed a borderline significant association with anxiety (*β* = 0.134, *p* = 0.06).

**Table 6:** Regression analyses of the hospital anxiety and depression scale scores for anxiety compared by disease-modifying antirheumatic drugs.

		Model 1			Model 2		
HADS score for anxiety		*β*	*p-*Value	95% CI	*β*	*p-*Value	95% CI
	Abatacept	0.023	0.790	−0.434 – 0.570	−0.020	0.813	−0.534 – 0.419
	Adalimumab	0.069	0.493	−0.260 – 0.537	0.054	0.574	−0.273 – 0.490
	Certolizumab	−0.051	0.547	−0.631 – 0.335	−0.050	0.533	−0.610 – 0.317
	Etanercept	−0.080	0.425	−0.603 – 0.255	−0.081	0.392	−0.581 – 0.229
	Hydroxychloroquine	0.038	0.663	−0.200 – 0.313	0.002	0.982	−0.241 – 0.246
	Infliximab	0.018	0.811	−0.765 – 0.976	−0.006	0.937	−0.861 – 0.794
	Leflunomide	0.158	0.037^*^	0.033–10.026	0.134	0.066^*^	−0.030 – 0.928
	Methotrexate	−0.035	0.735	−0.325 – 0.230	−0.069	0.481	−0.356 – 0.169
	Rituximab	0.041	0.633	−0.411 – 0.674	0.018	0.831	−0.464 – 0.576
	Secukinumab	−0.009	0.896	−1.467 – 1.286	−0.017	0.799	−1.473 – 1.135
	Sulfasalazine	−0.011	0.885	−0.672 – 0.580	−0.022	0.763	−0.689 – 0.507
	Tocilizumab	−0.063	0.422	−1.077 – 0.453	−0.086	0.253	−1.163 – 0.308
	Tofacitinib	0.030	0.682	−0.411 – 0.628	0.026	0.718	−0.405 – 0.586

CI, confidence interval; DMARDs, disease-modifying antirheumatic drugs; HADS, hospital anxiety and depression scale; ^*^p < 0.05 is statistically significant; Model 1, Regression analyses of DMARDs with anxiety; Model 2, Regression analyses of DMARDs with anxiety, adjusted for sociodemographic lifestyle factors, the use of combined DMARDs (using one of the traditional DMARDs in combination with one of the new DMARDs), and inflammatory markers; not taking the medication = reference category; β, standardized regression coefficient.

**Table 7:** Regression analyses of the hospital anxiety and depression scale scores for depression compared by disease-modifying antirheumatic drugs.

		Model 1			Model 2		
HADS score depression		*β*	*p-*Value	95% CI	*β*	*p-*Value	95% CI
	Abatacept	0.030	0.724	−0.371 – 0.533	−0.015	0.850	−0.468 – 0.387
	Adalimumab	−0.006	0.956	−0.369 – 0.349	−0.011	0.910	−0.362 – 0.323
	Certolizumab	−0.091	0.271	−0.679 – 0.192	−0.088	0.266	−0.651 – 0.181
	Etanercept	−0.186	0.061^*^	−0.755 – 0.018	−0.189	0.043^*^	−0.738 – −0.011
	Hydroxychloroquine	−0.036	0.673	−0.281 – 0.182	−0.075	0.353	−0.322 – 0.115
	Infliximab	0.014	0.849	−0.708 – 0.860	−0.017	0.813	−0.831 – 0.653
	Leflunomide	0.127	0.088	−0.058 – 0.836	0.100	0.159	−0.122 – 0.738
	Methotrexate	−0.112	0.273	−0.389 – 0.111	−0.146	0.132	−0.416 – 0.055
	Rituximab	0.051	0.552	−0.341 – 0.637	0.020	0.807	−0.409 – 0.525
	Secukinumab	0.027	0.701	−0.998 – 10.481	0.022	0.735	−0.969 – 1.371
	Sulfasalazine	−0.001	0.995	−0.565 – 0.562	−0.002	0.979	−0.544 – 0.529
	Tocilizumab	−0.059	0.448	−0.955 – 0.423	−0.072	0.326	−0.989 – 0.331
	Tofacitinib	0.052	0.474	−0.298 – 0.638	0.043	0.532	−0.303 – 0.585

CI, confidence interval; DMARDs, disease-modifying antirheumatic drugs; HADS, hospital anxiety and depression scale; ^*^p < 0.05 is statistically significant; Model 1: Regression analyses of DMARDs with depression; Model 2: Regression analyses of DMARDs with depression, adjusted for sociodemographic lifestyle factors the use of combined DMARDs (using one of the traditional DMARDs in combination with one of the new DMARDs), and inflammatory markers; not taking the medication = reference category; β, standardized regression coefficient.

Patients with RA taking etanercept showed significantly lower depression scores after adjusting for confounders (*β* = −0.189, value of *p* = 0.043) compared to patients who were not treated with etanercept (Table 7). The use of methotrexate, sulfasalazine, and tocilizumab showed lower scores for depression and anxiety; however, the association did not reach statistically significant levels (Tables 6, 7).

## 4. Discussion

To the best of our knowledge, this is the first study to examine the relationship between anxiety, depression, and DMARDs considering social determinants and lifestyle factors in the Saudi RA population. Our study showed a significant association between the HADS scores and exposure to the conventional DMARD leflunomide (selective inhibitor of *de novo* pyrimidine synthesis), biologic DMARD tocilizumab (IL-6 antagonist), and etanercept, a biologic tumor necrosis factor α (TNF-α) inhibitor, considering several clinical, sociodemographic, and lifestyle factors.

Few studies have examined the effect of conventional DMARDs on depression and anxiety among an RA population. Hydroxychloroquine is a common conventional DMARD used for treating RA, which has shown no psychiatric effects such as depression, anxiety, and suicide ideation (11, 17). In line with these studies, we did not find a significant relationship with the anxiety or depression scores and taking hydroxychloroquine.

However, we found a significant association between the HADS score for anxiety and depression and taking leflunomide (value of *p* = 0.006, value of *p* = 0.004, respectively). Patients with RA on leflunomide had significantly higher scores for anxiety (β = 0.158, value of *p* = 0.037) when compared to the patients who did not take leflunomide. After adjusting for the use of combined DMARDs, inflammatory markers, and sociodemographic and lifestyle factors, patients with RA treated with leflunomide showed a borderline significant association with anxiety; this finding could be underestimated as few patients in our study population used leflunomide. However, this finding should be interpretated with caution as we had only 2 patients with RA, who were on leflunomide; we suggest further investigation into this finding in future studies. In contrast to our finding, a study in Brazil reported patients treated with leflunomide had the lowest average HADS score for both, depression and anxiety. However, they did not examine social determinants of RA, and it was a cross-sectional study where the causal relationship between RA and the presence of depression and anxiety cannot be inferred (11). In our study, these sociodemographic and lifestyle factors were adjusted in the regression models to eliminate their effects on the association between exposure to DMARDs and depression and anxiety scores.

Although the new biologic DMARDs decreased depression symptoms in patients with RA (18), the available evidence is still contradictory as a different study reported enhanced patient mood. The mechanism of this observed effect of the new DMARDs could be due to the direct effect of suppressing the inflammation and resolution of pain or possibly acting as an antidepressant (12). Depression symptoms decreased dramatically after initiating these therapies for patients with RA (19). Lower anxiety and depression were associated with enhanced fatigue scores after treatment with tocilizumab (20). Another study used the HADS to evaluate the quality of life of patients with RA taking tocilizumab and concluded that there was some decrease in depression, anxiety, and fatigue symptoms (21). However, a recent study reported a controversial finding that taking biologic DMARDs did not decrease and could worsen depression, pain, sleep, and anxiety (22); however, it was conducted on a population undergoing immunocompromised hematopoietic cell transplantation.

The coexistence of inflammation in autoimmune diseases (such as RA) and depression has been established (23), although the exact mechanism is still not fully understood. One of the possible explanations is the existence of shared intimate pathophysiological mechanisms between peripheral and brain immune responses; this association includes the negative effect of pro-inflammatory cytokines on monoaminergic neurotransmission and neurotrophic factors (23, 24). IL-6 levels play a pivotal role in the relationship between inflammatory disorders, depression, and anxiety, where low levels of IL-6 are associated with anxiety and high levels are associated with depression (25–27). In a recently conducted study, RA patients who had depression and anxiety showed higher scores of RA severity index such as disease activity score on 28 joints (DAS-28). It also found that RA patients with depression and anxiety had a higher level of immune inflammatory markers in comparison with RA patients with no mood disorders. These markers include CRP and IL-6; the study concluded that depression and anxiety in RA patients were driven by the same immune-inflammatory pathway which is part of the pathophysiology of RA disease itself, and not driven by the experience and burden of the disease (28). With these findings being noted, multiple papers in literature have shown that medications that target inflammatory cytokines have a positive effect on depression and anxiety (25–27). Regarding the beneficial effect of an IL-6 antagonist (i.e., tocilizumab), a large multicenter study found that weekly injections of tocilizumab over 24 weeks had a favorable effect in decreasing anxiety and depression levels (29). A cohort study in the US assessed the impact of tocilizumab monotherapy and found it ameliorated the effect of both, depression and anxiety by 33% (13). In line with these findings in the literature, we found that there was a significant inverse association between the HADS score for anxiety and taking tocilizumab (lower scores for depression and anxiety) (*p* = 0.009). However, this association was insignificant after adjusting for use of combined DMARDs, inflammatory markers, and sociodemographic and lifestyle factors.

Several studies have shown that patients with rheumatic diseases could benefit from TNF-α antagonist (etanercept) treatment for depression (29–34). In line with our result, patients treated with etanercept had lower levels of depression compared to those taking other DMARDs (*β* = −0.189, value of *p* = 0.043). However, further studies are needed to validate the potential benefit of a TNF-α antagonist for treatment of depression in patients with RA, which could be driven by inflammatory cytokines. Although most of the studies line up with the fact that RA is linked with depression and anxiety, there is scarce evidence that shows the interaction and effect of RA in relation to the medication used by the patients with RA. Thus, available evidence supporting the association between pro-inflammatory cytokines and depression and anxiety indicates that some of the DMARDs can provide benefits to mental health independent of improvements in the RA disease score.

### 4.1. Strengths and limitations

Our findings have important implications for ultimately reducing the economic impact on the healthcare system and improving routine clinical outcomes. Another strength of this study is that the participants were selected from outpatient clinics at the KKUH, which is one of the largest tertiary hospitals in Saudi Arabia that accepts referrals for all age groups across Saudi Arabia and provides medical access to all patients with suspected RA. Participants were classified into levels of anxiety and depression according to their HADS scores using a standardized validated scoring tool (HADS-A 8 or HADS-D 8, respectively) (14). Depression and anxiety scores were compared to determine factors that influence RA patients’ levels of depression and anxiety, particularly the type of DMARDs. This study also adjusted for common confounders, including clinical history, medications and the use of combined DMARDs, sociodemographic and lifestyle factors.

Our study has some limitations. This is a cross-sectional study; therefore, we cannot have a causal conclusion. Due to the observational nature of our study, we could not measure diseases activity score such as DAS-28; the data was collected from the hospital database and then, the electronic anxiety depression questionnaire was sent to the eligible RA patients. Hence, no face-to-face interview was conducted to perform the clinical examination and to calculate the DAS-28 score. It should be highlighted that this study is the first cross-sectional (preliminary study) in preparation for the future longitudinal prospective cohort study where the patients will be assessed clinically, the disease activity score will be calculated, and RA patients will be compared based on their disease’s severity. We also suggest that further clinical trials are conducted to examine the influence of different types of DMARDs on the development of depression and anxiety over time in patients with RA. Respondent fatigue from overexposure to surveys was highly expected; this phenomenon is challenging as it negatively affects the response rate (35).

## 5. Conclusion

The present study reported the prevalence of anxiety and depression among patients with RA and showed that the level of these psychological disorders may differ between patients with RA depending on the type of DMARDs used. Assessment of this relationship could help physicians select less aggressive treatments and alert the treating physician to the possibility of patients developing comorbid psychiatric conditions during treatment. The current study showed that psychiatric aspects such as depression, anxiety, and suicide ideation may differ between groups of patients with RA on different drugs and the importance of considering these factors in therapeutic decisions. The co-occurrence of depression in patients with RA warrants regular screening and monitoring.

## Data availability statement

The original contributions presented in the study are included in the article/Supplementary material, further inquiries can be directed to the corresponding author.

## Ethics statement

The studies involving human participants were reviewed and approved by Institutional Review Board of King Saud University College of Medicine (Ethics Approval Number: E-22-6743). The patients/participants provided their written informed consent to participate in this study.

## Author contributions

LB contributed as the principal investigator and worked on planning and execution of the overarching research goals and aims, supervised designing the methodology, study materials, and computing process, rewrote the manuscript draft, interpreted the data and computational techniques of the data analysis, did a critical review, commentary, and revision of the manuscript, and ensured the manuscript descriptions are accurate and agreed upon by all authors. MKA, FA, AA, AS, and MTA conducted the research and investigation process, performed the data collection and curation, implemented the data coding and cleaning, application of statistical software, and computational techniques of the data analysis, and wrote the first draft of the manuscript. All authors contributed to the article and approved the submitted version.

## Conflict of interest

The authors declare that the research was conducted in the absence of any commercial or financial relationships that could be construed as a potential conflict of interest.

## Publisher’s note

All claims expressed in this article are solely those of the authors and do not necessarily represent those of their affiliated organizations, or those of the publisher, the editors and the reviewers. Any product that may be evaluated in this article, or claim that may be made by its manufacturer, is not guaranteed or endorsed by the publisher.

## Abbreviations

DMARDs: disease-modifying antirheumatic drugs
HADS: Hospital Anxiety and Depression Scale
IL: interleukin
KKUH: King Khalid University Hospital
RA: rheumatoid arthritis
