# Supplementary material for: Effect of type of disease-modifying antirheumatic drugs on depression and anxiety of patients with rheumatoid arthritis in Saudi Arabia: a cross-sectional study
Source: Front Psychiatry. 2023 Jun 6;14:1184720. doi: 10.3389/fpsyt.2023.1184720 (PMC10280020; doi:10.3389/fpsyt.2023.1184720)
Supplement: Supplementary file 1 [file Data_Sheet_1.pdf]

For REC use only:
Expedited [ ]
Proposal No. _____

INFORMED CONSENT FOR ONLINE QUESTIONNAIRE-BASED SURVEY
Form # KSU-REC 006OS-E

King Saud University, Riyadh, Kingdom of Saudi Arabia

Dear ***Participant***,

Thank you for agreeing to take part in this online questionnaire survey with a study title of: Depression And Anxiety In Rheumatoid Arthritis Patients and the DMARDs In King Khalid University Hospital.
The purpose of this online survey is to help filling the gap in the published literature on the Depression And Anxiety In Rheumatoid Arthritis Patients and the DMARDs.

It will take approximately 5-10 minutes to complete the survey.
Be assured that all answers you provide will be kept in a strictest confidentiality. Please feel free to call or text Dr. Leena Baghdadi, Mobile No. 0501235269 to answer your questions.

If you are willing to participate this online survey, please click "***Next***" to begin.

This document is copyright © (KSU-REC) King Saud University, 2017. No part may be reproduced in any form or by any means, or transmitted, or published without prior written consent from King Saud University.

نموذج موافقه مسبقة على استبيان الكتروني

رقم الاستمارة: KSU-REC 006OS-E

عزيزتي المشاركة/ عزيزي المشارك،

شكرًا على موافقتك على المشاركة في هذا الاستبيان الالكتروني لدراسة بعنوان **الاكتئاب والقلق عند مرضى الالتهاب الروماتيدي في مستشفى الملك خالد الجامعي.**

والغرض من هذا الاستبيان الالكتروني هو تقدير مدى انتشار اضطراب القلق والاكتئاب بين المرضى المصابين بالاتهاب الروماتيدي والذين يرتادون مستشفى جامعة الملك خالد. وسيستغرق اكمال هذا الاستبيان حوالي 10-5 دقيقة.

نؤكد لك أن جميع إجاباتك التي ستقدمها ستبقى في سرية تامة، لا تترددي في الاتصال بالدكتورة لينة بغدادي أو ارسال رسالة نصية، على الرقم 0501235269 للإجابة على أسئلتك.

إذا كنت موافق على المشاركة في هذا الاستبيان الالكتروني فيرجى النقر على "التالي" للبدء.

This document is copyright © (KSU-REC) King Saud University, 2017. No part may be reproduced in any form or by any means ,or transmitted, or published without prior written consent from King Saud University.
