# Supplementary material for: Effect of type of disease-modifying antirheumatic drugs on depression and anxiety of patients with rheumatoid arthritis in Saudi Arabia: a cross-sectional study
Source: Front Psychiatry. 2023 Jun 6;14:1184720. doi: 10.3389/fpsyt.2023.1184720 (PMC10280020; doi:10.3389/fpsyt.2023.1184720)
Supplement: Supplementary file 3 [file Data_Sheet_3.pdf]

Supplementary Material

Effect of Type of DMARDs on Depression and Anxiety of Patients with Rheumatoid Arthritis in Saudi Arabia: A Cross-sectional Study

Leena R Baghdadi[1*], Mohammed K Alhassan[2], Fawaz H Alotaibi[2], Anas A Alsuwaida[2], Ali E Shehadah[2], Mohammed T Alzahrani[2]

*** Correspondence:** Dr Leena R. Baghdadi lbaghdadi@ksu.edu.saemail@uni.edu

Table S1. Post-Hoc of Chi-Square Test on DMARDs comparing depression and anxiety of patients by disease-modifying antirheumatic drugs

Disease-modifying antirheumatic drugs	Pairwise Comparison	P-Value	
		Depression	Anxiety
Abatacept	Normal Vs Borderline Normal	0.076	0.530
	Normal Vs Abnormal	1.000	1.000
	Borderline Normal Vs Abnormal	0.246	0.540
Adalimumab	Normal Vs Borderline Normal	0.820	0.470
	Normal Vs Abnormal	0.730	0.230
	Borderline Normal Vs Abnormal	0.570	1.000
Certolizumab	Normal Vs Borderline Normal	0.860	1.000
	Normal Vs Abnormal	0.290	0.900
	Borderline Normal Vs Abnormal	0.220	0.990
Etanercept	Normal Vs Borderline Normal	0.260	0.520
	Normal Vs Abnormal	0.310	0.270
	Borderline Normal Vs Abnormal	1.000	1.000
Hydroxychloroquine	Normal Vs Borderline Normal	0.920	0.500
	Normal Vs Abnormal	0.840	0.970
	Borderline Normal Vs Abnormal	1.000	0.490
Infliximab	Normal Vs Borderline Normal	1.000	1.000
	Normal Vs Abnormal	0.220	0.360
	Borderline Normal Vs Abnormal	0.340	0.720
Leflunomide	Normal Vs Borderline Normal	0.631	0.963
	Normal Vs Abnormal	0.004*	0.016*
	Borderline Normal Vs Abnormal	0.216	0.128
Methotrexate	Normal Vs Borderline Normal	0.550	0.510
	Normal Vs Abnormal	0.250	0.750
	Borderline Normal Vs Abnormal	0.730	0.860
Rituximab	Normal Vs Borderline Normal	0.720	1.000
	Normal Vs Abnormal	0.620	0.940
	Borderline Normal Vs Abnormal	0.400	1.000
Secukinumab	Normal Vs Borderline Normal	0.600	1.000
	Normal Vs Abnormal	-	1.000
	Borderline Normal Vs Abnormal	1.000	-
Sulfasalazine	Normal Vs Borderline Normal	0.510	1.000
	Normal Vs Abnormal	0.820	1.000
	Borderline Normal Vs Abnormal	0.340	1.000
Tocilizumab	Normal Vs Borderline Normal	0.690	0.022*
	Normal Vs Abnormal	1.000	0.520
	Borderline Normal Vs Abnormal	0.870	-
Tofacitinib	Normal Vs Borderline Normal	0.410	0.780
	Normal Vs Abnormal	0.630	1.000
	Borderline Normal Vs Abnormal	1.000	0.116
Combined medication	Normal Vs Borderline Normal	0.220	1.000
	Normal Vs Abnormal	1.000	1.000
	Borderline Normal Vs Abnormal	0.320	1.000

*p value < 0.05

Table S2. Post-Hoc of Chi-Square Test on DMARDs comparing depression and anxiety of rheumatoid arthritis patients by their chronic diseases

Chronic Disease	Pairwise Comparison	P-Value	
		Depression	Anxiety
High blood pressure	Normal Vs Borderline Normal	0.630	0.930
	Normal Vs Abnormal	0.990	0.150
	Borderline Normal Vs Abnormal	0.640	0.530
Type 2 diabetes mellitus	Normal Vs Borderline Normal	0.180	0.065
	Normal Vs Abnormal	0.650	0.932
	Borderline Normal Vs Abnormal	0.120	0.142
Dyslipidemia	Normal Vs Borderline Normal	1.000	0.240
	Normal Vs Abnormal	0.180	0.500
	Borderline Normal Vs Abnormal	0.290	0.730
Cardiovascular diseases	Normal Vs Borderline Normal	0.140	0.766
	Normal Vs Abnormal	0.220	0.087
	Borderline Normal Vs Abnormal	1.000	0.691
Autoimmune diseases	Normal Vs Borderline Normal	0.022*	0.664
	Normal Vs Abnormal	0.001*	0.035*
	Borderline Normal Vs Abnormal	0.392	0.470

*p value <0.05

Table S3. Post-Hoc of Chi-Square Test comparing depression and anxiety of rheumatoid arthritis patients by their medications (other than disease-modifying antirheumatic drugs)

Medication	Pairwise Comparison	P-Value	
		Depression	Anxiety
Hypertension medication	Normal Vs Borderline Normal	0.400	0.677
	Normal Vs Abnormal	0.850	0.061
	Borderline Normal Vs Abnormal	0.370	0.534
Cardiac disease medication	Normal Vs Borderline Normal	0.780	0.880
	Normal Vs Abnormal	0.970	1.000
	Borderline Normal Vs Abnormal	0.720	1.000
Immuno-suppressive medication	Normal Vs Borderline Normal	0.005*	0.800
	Normal Vs Abnormal	0.057	0.340
	Borderline Normal Vs Abnormal	0.856	0.960
Acne medication	Normal Vs Borderline Normal	0.600	-
	Normal Vs Abnormal	-	0.600
	Borderline Normal Vs Abnormal	1.000	1.000
Oral contraceptive pills	Normal Vs Borderline Normal	1.000	0.410
	Normal Vs Abnormal	1.000	0.940
	Borderline Normal Vs Abnormal	1.000	0.810
Nonsteroidal anti-inflammatory drugs	Normal Vs Borderline Normal	0.925	0.380
	Normal Vs Abnormal	0.019*	0.070
	Borderline Normal Vs Abnormal	0.079	0.820
Cancer treatment	Normal Vs Borderline Normal	0.960	1.000
	Normal Vs Abnormal	0.620	0.270
	Borderline Normal Vs Abnormal	1.000	0.520

*p value <0.05
